# Taxonomic notes on the *Macrocheilus* Hope (Coleoptera, Carabidae, Helluonini) from Oriental Region, with description of one new species from the Philippines

**DOI:** 10.3897/zookeys.228.3401

**Published:** 2012-10-18

**Authors:** Danyang Zhao, Mingyi Tian

**Affiliations:** 1 Guangdong Academy of Forestry, Guangzhou, Guangdong 510520, China; 2 Department of Entomology, College of Natural Resources and Environment, South China Agricultural University, Guangzhou, Guangdong 510640, China

**Keywords:** Coleoptera, Carabidae, Helluonini, *Macrocheilus*, Oriental Region, new species

## Abstract

Taxonomic notes, together with illustrated characters, on the oriental species of the genus *Macrocheilus* Hope, 1838 (Carabidae, Helluonini) are provided. One new species, *Macrocheilus deuvie* **sp. n.** is described from the Philippines. A key to all species of *Macrocheilus* in the Oriental Region is also provided.

## Introduction

The genus *Macrocheilus* Hope, 1838 is composed of Oriental, Palaearctic and Ethiopian species (Csiki 1932, Reichardt 1974). In total, fifty-six species of the genus are reported, among them, thirty-five species in the Ethiopian Region, nineteen in Oriental Region, and only two in Palaearctic Region (Lorenz 2005, Hůrka 2003, Zhao and Tian 2010).

For the Oriental *Macrocheilus*, Heller (1900) gave a table of ten species. But of them, two (*dorsalis* Klug and *scapularis* Klug) are actually African species, one (*distactus* Wiedemann) belongs to the genus *Creagris*, and one (*tripustulatus* Fairmaire) is a species of *Pheropsophus*. Andrewes (1920) dealt with ten Oriental species of the genus. Jedlička (1963) treated the East Asian *Macrocheilus* species and mentioned only five species. Park et al. (2006) listed six species from Vietnam. Zhao and Tian (2010) described seven new species and gave a key to Chinese species.

The aim of this paper is to provide taxonomic notes on all Oriental species of *Macrocheilus* by means of checking most of the type materials and a few other materials. As a result of the study, the examined materials are preserved in the Museum National d’Histoire Naturelle, Paris, France (MNHN). One new species of *Macrocheilus* from the Philippines is described. In addition, a distribution map of the genus in the Oriental Region is provided.

## Materials and methods

Materials for this study were dry mounted specimens. Dissection of specimens was done following the standard technique described by Lindroth (1974). Observations and measurements were made under stereo microscope (Leica, MZ125) and ocular microscope (Wild M5).

Abbreviations for the collections in which the type materials are deposited as follows:

**LMN** Leiden Museum, the Netherlands

**MDSG** Museum Dresden, Saxony, Germany

**MGI** Museum Genoa, Italy

**MNHN** Museum National d’Histoire Naturelle, Paris, France

**NHML** Natural History Museum, London, the U.K.

**NMP** National Museum Prague, Czech Republic

**SCAU** South China Agricultural University, Guangzhou, China

**SNSD** Staatliches Naturhist. Sammlungen Museum für Tierkund, Dresden, Germany

**ZMUC** Zoological Museum University of Copenhagen, Copenhagen, Denmark

## Taxonomic treatment

### Genus Macrocheilus

Hope, 1838

http://species-id.net/wiki/Macrocheilus

Macrocheilus Hope 1838: 166. Type species: *Macrocheilus bensoni* Hope, 1838. Jeannel 1949: 1041. Zhao and Tian 2010:4.
Acanthogenius Reiche 1842: 334. Type species: *Helluo impictus* Wiedemann, 1823.

#### Generic diagnosis.

Medium sized (length from 8.0 to 17.0 mm), elongate, whole body coarsely punctuate and pubescent, except for labrum and middle region of ventral side of head. Head with two supraorbital setae on each side; ligula fairly wide, deeply impressed beneath at sides of base; mentum deeply emarginated, with a long and slender tooth; palpi varied in form; labrum extraordinarily or well developed; mandibles dorsally covered by labrum or not covered; antennae stout and flat, densely pubescent from antennomere 5. Pronotum truncate-cordiform or quadrate, basal margin more or less produced backwards in middle; lateral margin with two setae, one just before middle, and the other at hind angle. Elytra with deep striae and setiferous pores; intervals slightly or rather convex, interval 8 usually wide. Metepisterna elongate and very narrow in all species. Tarsomere 4 emarginate. Wings fully developed. No externally visible sexual dimorphic characters present. Median lobe of aedeagus varied in form. Parameres of aedeagus quite similar, elongate, the left one larger than the right one.

#### Differences with other genera of Helluonini in the Oriental Region.

*Macrocheilus* spp., with larger body size, simple 4^th^ tarsomeres, and non-securiform labial palpomere are distinctly different from *Colfax* spp. (smaller body size, securiform labial palpomere) and *Creagris* spp. (bilobed 4^th^ tarsomere; smaller body size). Functional wings and the occurrence of a long spiniform median tooth of mentum of *Macrocheilus* distinguish them from *Omphra* spp. (which are brachypterous and have a short median tooth of the mentum).

#### Generic range.

Oriental Region (China, Vietnam, Laos, Cambodia, Myanmar, India, Sri Lanka, the Philippines, Malaysia and Indonesia), Palaearctic Region (Pakistan, Syria, Asia Minor), Ethiopian Region (Ethiopia, Tanzania, Uganda, Senegal, Guinea, Guinea-Bissau, Sierra Leone, Togo, Nigeria, Central Africa, Cameroon, Gabon, Congo, Zimbabwe, Angola, South Africa, Madagascar).

#### Key to species of Oriental *Macrocheilus*

**Table:** 

1	Elytra without spots	2
–	Elytra spotted	3
2	Head and pronotum red; ligula with apical outer angles rounded; apex of lateral lobes of mentum fairly acute (Fig. 9); labrum with apical margin not sinuate (Fig. 1). Length 6.5 mm	*Macrocheilus bicolor* Andrewes
–	Whole body piceous; ligula with apical outer angles rectangular; apex of lateral lobes of mentum rounded (Fig. 10); labrum with apical margin plurisinuate (Fig. 2). Length 15.0 mm	*Macrocheilus impictus* (Wiedemann)
3	Maxillary palpomere 4 not flattened and dilated, lateral lobes of mentum rounded on outer margin, mandibles not exposed, lateral margin of labrum rounded, front margin of clypeus not or slightly emarginate in middle, elytron with one or two spots	4
–	Maxillary palpomere 4 strongly flattened and dilated, lateral lobes of mentum sinuate behind middle or on anterior one-third, mandibles exposed, lateral margin of labrum distinctly sinuate on anterior one-third, front margin of clypeus deeply and widely emarginate in middle, elytron with one spot	16
4	Elytron with one spot	5
–	Elytron with two spots	10
5	Labrum with anterior setae distinctly on upper surface margin	6
–	Labrum with anterior setae close to or on the apical margin	8
6	Labrum with apex narrow (Fig. 3); mentum with median tooth stout and wide, strongly sinuate at middle of lateral margins (Fig. 11). Length 25.0 mm	*Macrocheilus immanis* Andrewes
–	Labrum with apex wide; mentum with median tooth slender, almost straight at sides	7
7	Elytral spot transverse and almost rectangular (Fig. 46); median tooth of mentum with four or five setae (Fig. 12). Length 16.0 mm	*Macrocheilus niger* Andrewes
–	Elytral spot cruciform (Fig. 47); median tooth of mentum with two setae. Length 14.5 mm	*Macrocheilus asteriscus* (White)
8	Elytral spot rounded, maxillary palpomere 4 roundly elongate; anterior setae of labrum close to apical margin. Length 12.5–14.0 mm	*Macrocheilus vitalisi* Andrewes
–	Elytral spot oblong, maxillary palpomere 4 short and stout (Fig. 21); anterior setae of labrum along or on apical margin	9
9	Elytral spot covering intervals 3–7, anterior seta of labrum on apical margin (Fig. 5). Length 13.0 mm	*Macrocheilus binotatus* Andrewes
–	Elytral spot covering intervals 2–7, anterior seta of labrum along apical margin. Length 10.0 mm	*Macrocheilus macromaculatus* Louwerens
10	Labrum with anterior setae on apical margin; ligula thickened at apex	11
–	Labrum with anterior setae beneath apex; ligula thin at apex	13
11	Labrum with apex wide; mentum with median tooth sinuate near apex on lateral margin, lateral lobes obtuse at apex. Length 15.5–17.0 mm	*Macrocheilus gigas* Zhao & Tian
–	Labrum with apex pointed; mentum with median tooth not sinuate on lateral margin, lateral lobes sharp at apex	12
12	Body length 11.7–11.0 mm; elytral spots smaller (Fig. 51); labrum exceptionaly convex on anterior portion which results apex can not be seen; maxillary palpomere 4 strongly and roundly dilated on anterior half portion	*Macrocheilus parvimaculatus* Zhao & Tian
–	Body length 12.0–12.5 mm; elytral spots larger (Fig. 52); labrum normally convex, apex visible (Fig. 6); maxillary palpomere 4 slightly dilated (Fig. 22)	*Macrocheilus tripustulatus* (Dejean)
13	Maxillary palpomere 4 strongly dilated	14
–	Maxillary palpomere 4 not or slightly dilated	15
14	Head and pronotum reddish brown (Fig. 53); labrum relatively long; pronotum elongate; tibiae testaceous. Length 8.4 mm	*Macrocheilus chaudoiri* Andrewes
–	Head and pronotum black; labrum short (Fig. 7); pronotum wide; tibiae black. Length 8.0 mm	*Macrocheilus nigrotibialis* Heller
15	Maxillary palpomere 4 slightly dilated; labrum not recurved at apex; clypeus with irregular setae on middle. Length 15.0 mm	*Macrocheilus bensoni* Hope
–	Maxillary palpomere 4 not dilated (Fig. 24); labrum curved at anterior part (Fig. 8); clypeus almost glabrous on middle. Length 9.5 mm	*Macrocheilus deuvie* sp. n.
16	Elytral spots large (1.5~2.2 mm), nearly rectangular	17
–	Elytral spots small (1.3~1.4 mm), not rectangular	19
17	Color brown; labrum with only three pairs of labral setae, apex pointed rounded; mandibles obtuse at apices; median tooth of mentum with lateral margin not sinuate. Length 10.8 mm	*Macrocheilus fuscipennis* Zhao & Tian
–	Color black; labrum with four pairs of labral setae, apex widely rounded; mandibles sharp at apices; median tooth of mentum with lateral margin sinuate	18
18	Labrum with additional setae located between the anterior and intermediate setae (Fig. 8 in Zhao and Tian 2010); median tooth of mentum with lateral margin sinuate in middle; mandibles less sharp at apices. Length 12.3–12.5 mm	*Macrocheilus solidipalpis* Zhao & Tian
–	Labrum with additional setae located before the anterior setae (Fig. 9 in Zhao and Tian 2010); median tooth of mentum with lateral margin sinuate on anterior one-third; mandibles sharper at apices. Length 11.0 mm	*Macrocheilus cheni* Zhao & Tian
19	Labrum with anterior and intermediate setae distance from each other, apex pointed rounded (Fig. 10 in Zhao and Tian 2010); mandibles sharp at apices; median tooth of mentum sharp at apex; pronotum almost quadrate. Length 12.1 mm	*Macrocheilus quadratus* Zhao & Tian
–	Labrum with anterior and intermediate setae closed each other, apex widely rounded (Fig. 11 in Zhao and Tian 2010); mandibles obtuse at apices; median tooth of mentum strongly obtuse at apex; pronotum nearly cordiform. Length 11.7 mm	*Macrocheilus sinuatilabris* Zhao & Tian

### Macrocheilus bicolor

Andrewes, 1920

http://species-id.net/wiki/Macrocheilus_bicolor

**Figures 1–8.**
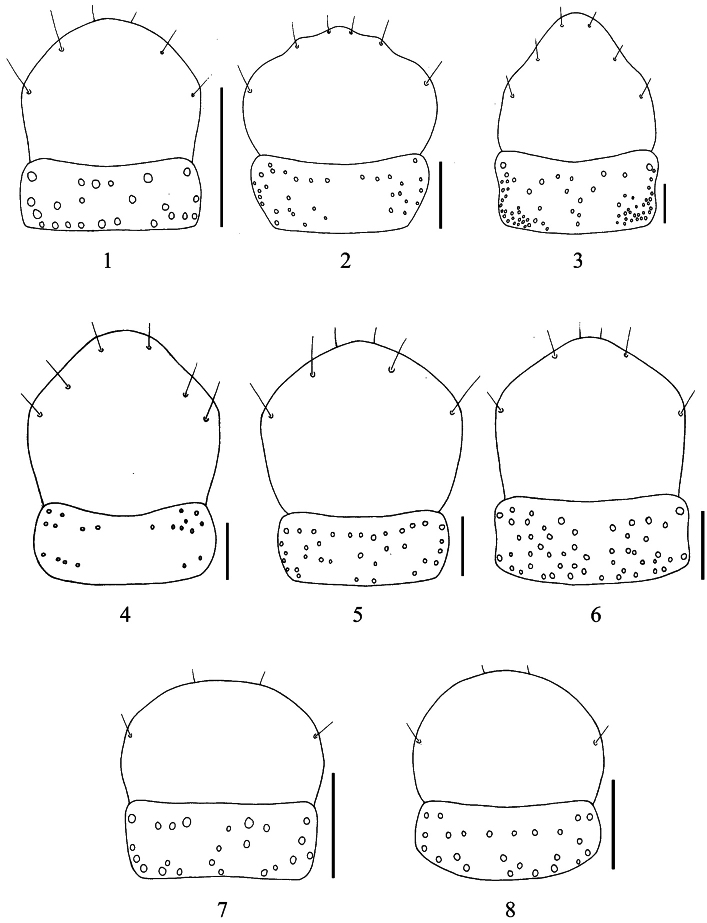
Labrum and clypeus of *Macrocheilus* (dorsal view): **1** *Macrocheilus bicolor* (holotype) **2** *Macrocheilus impictus* (male) **3** *Macrocheilus immanis* (holotype) **4** *Macrocheilus niger* (holotype) **5** *Macrocheilus binotatus* (female) **6** *Macrocheilus tripustulatus* (holotype) **7** *Macrocheilus nigrotibialis* (holotype) **8** *Macrocheilus deuvie* (holotype). Scale bar: 0.5 mm.

**Figures 9–24.**
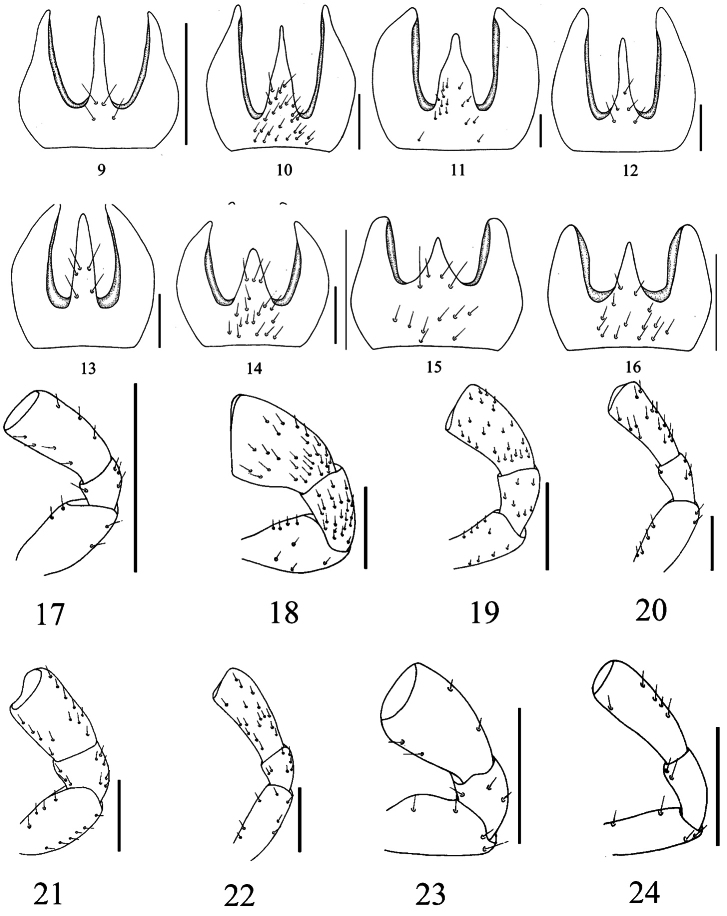
Mentum and Left maxillary palpi of *Macrocheilus* (ventral view): **9, 17** *Macrocheilus bicolor* (holotype) **10, 18** *Macrocheilus impictus* (male) **11, 19** *Macrocheilus immanis* (holotype) **12, 20** *Macrocheilus niger* (holotype) **13, 21** *Macrocheilus binotatus* (female) **14, 22** *Macrocheilus tripustulatus* (holotype) **15, 23** *Macrocheilus nigrotibialis* (holotype) **16, 24** *Macrocheilus deuvie* (holotype). Scale bar: 0.5 mm.

Figs 1 , 9, 17 , 37 , 43

Macrocheilus bicolor Andrewes 1920: 503; Andrewes 1930: 206; Csiki 1932: 1573; Lorenz 2005: 512. Type locality: India (Bombay: Belgaum), deposited in NHML.

#### Diagnosis.

Length 6.3–6.5 mm, width 2.5 mm. Head and prothorax red; elytra black or sometimes bluish black. Labrum (Fig. 1) semicircular in front, shortly depressed towards base, front pair of setae small and closely placed along the front margin, intermediate one at a distance from margin; ligula rectangular, with a wide and deep median impression, a pair of setae at a distance form apex depressed at base; apex truncate, outer apical angles rounded; mentum (Fig. 9) glabrous at base, both tooth and lobes elongate, slender and sharp at apex; tooth almost as long as lobes, two pairs of setae on base; lobes sinuate at a distance from apex along outer margin; maxillary palpi (Fig. 17) not dilated. Elytra without spots.

Female genitalia. Gonocoxite short, stout, inner margin arcuate and not sinuate, apex short and sharp (Fig. 37).

**Figures 25–42.**
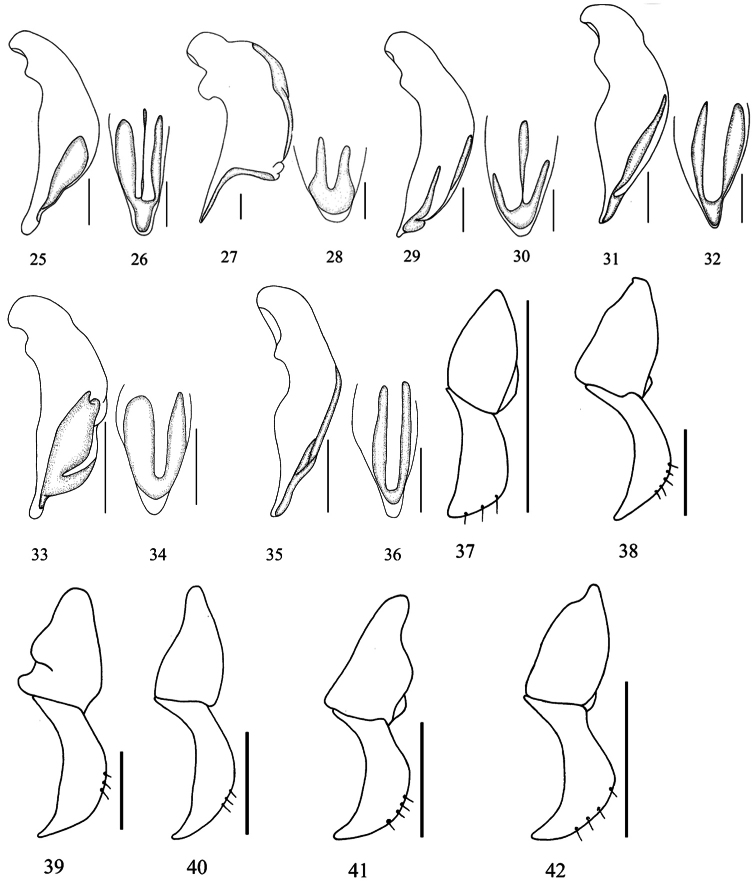
Aedeagus of *Macrocheilus* (left lateral and dorsal view) and Left gonopods of female genitalia (dorsal view): **25, 26, 38** *Macrocheilus impictus* **27, 28** *Macrocheilus immanis* (holotype) **29, 30, 39** *Macrocheilus niger* **31, 32, 41** *Macrocheilus tripustulatus* **33, 34, 42** *Macrocheilus nigrotibialis* (holotype) **35, 36** *Macrocheilus deuvie* (holotype) **37** *Macrocheilus bicolor* (holotype) **40** *Macrocheilus binotatus*. Scale bar: 0.5 mm.

**Figures 43–51.**
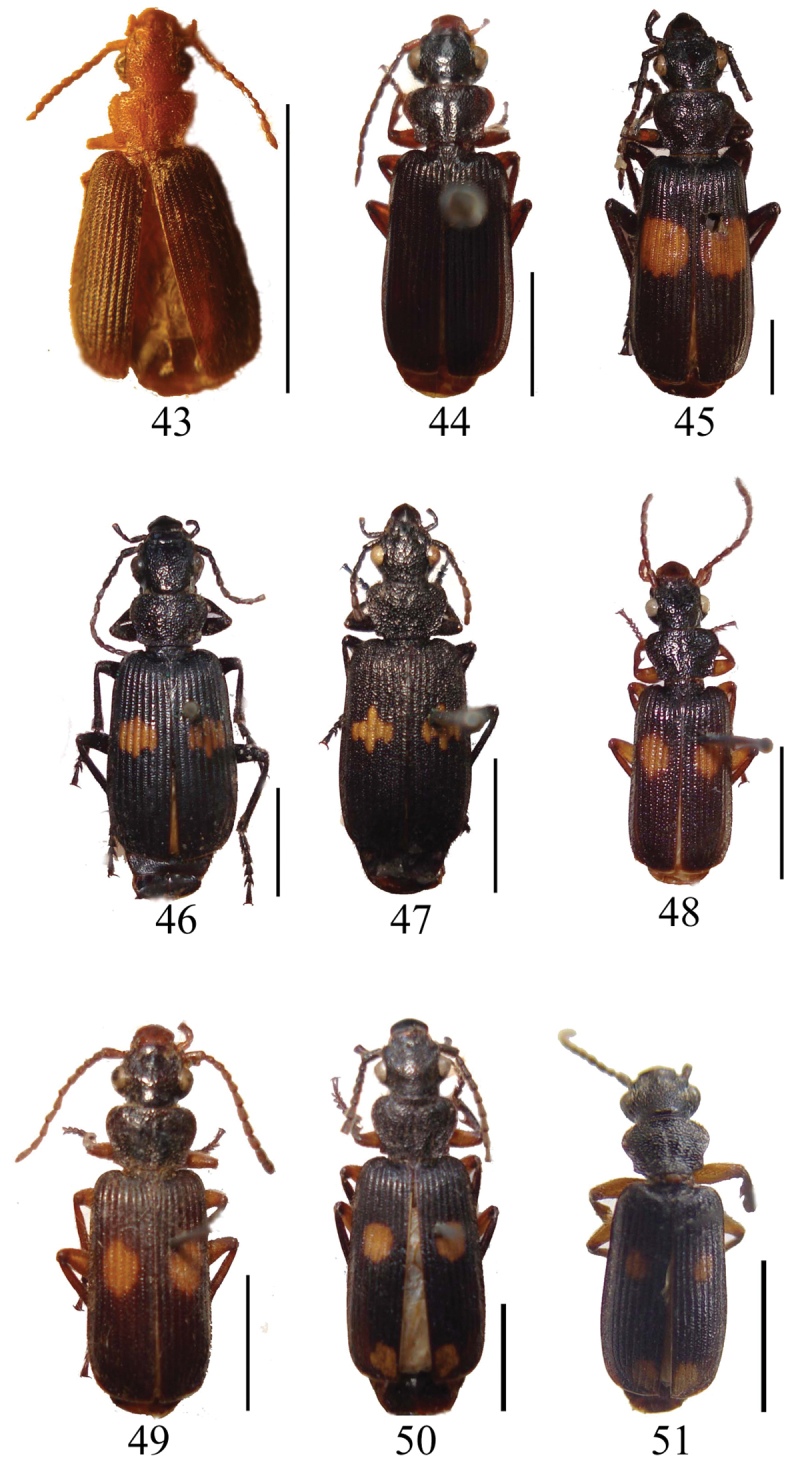
Habiti of *Macrocheilus* (dorsal view): **43** *Macrocheilus bicolor* (holotype) **44** *Macrocheilus impictus* (male) **45** *Macrocheilus immanis* (holotype) **46** *Macrocheilus niger* (holotype) **47** *Macrocheilus asteriscus* (male) **48** *Macrocheilus vitalisi* (holotype) **49** *Macrocheilus binotatus* (female) **50** *Macrocheilus gigas* (holotype) **51** *Macrocheilus parvimaculatus* (holotype).

#### Remarks.

This species is allied to the next species, *Macrocheilus impictus* (Wiedemann). Both differ from other species in the absence of elytral spot.

#### Materials examined.

Holotype: 1 female, labeled “Kaoeqaoe, 740, Type, *Macrochilus bicolor* Type Andr., H. E. Andrewes det.; H. E. Andrewes Coll. B. M. 1945–97.; *Macrochilus bicolor* Andrewes, 1920”, deposited in NHML. 1 ex, sex unknown (the abdomen missing), labeled “Bangalore, Chikkangalur, Taboourel, 1900; *Macrocheilus bicolor* Andrewes, H. E. Andrewes det.”, deposited in MNHN.

#### Distribution.

India.

### Macrocheilus impictus

(Wiedemann, 1823)

http://species-id.net/wiki/Macrocheilus_impictus

Figs 2 , 10, 18 , 25, 26, 38 , 44

Helluo impictus Wiedemann 1823: 49. Dejean 1825: 287; Reiche 1842: 335; Heller 1900: 3; Andrewes 1920: 503; 1921: 168; Csiki 1932: 1574; Lorenz 2005: 512. Type locality: India, deposited in ZMUC.

#### Diagnosis.

Length 14.5-15.0 mm, width 5.0-5.5 mm. Black. Labrum (Fig. 2) with front margin rounded and bisinuate, three pairs of setae close to margin, and front two pairs on sinuated area; mandibles slightly obtuse at apex; mentum (Fig. 10) irregularly setose in basal half, both tooth and lobes elongate and sharp at apex, tooth slightly shorter than lobes; maxillary palpomere 4 (Fig. 18) cylindrical and gradually dilated toward apex, rounded and obliquely truncate at apex. Elytra without spots.

Male genitalia. Median lobe dilated on dorsal side in middle partion, sinuate on ventral side; apical lamella elongate, not sinuate near apex, rounded at apex (Figs 25–26).

Female genitalia. Gonocoxite slender, five setae on dorsal surface, sharp at apex (Fig. 38).

#### Remarks.

Similar to *Macrocheilus bicolor* without elytral spots, distinctly differs by larger size, body black, sinuate front margin of labrum and plurisetose mental tooth.

#### Materials examined.

1 male, labeled “Indes Orientales, M^ts^ Kodeicanel, J. Castets 1886”; 1 male, “India, Bangalore. P.S. Nathan, 1936”; 1 male, “Punjab Baddia (Indes Angl.); G. Babault Avril 1914”; 1 male, “S. India, Medungadu, P.S. Nathan. 1936”; 3 males, “Java”, “Ex-Musaeo Chaudoir from Coll. Dejean”; 1 female, “Java”, “Ex-Musaeo Mniszech from Coll. Dejean”. All deposited in MNHN.

#### Distribution.

India (East India) and Indonesia (Java).

### Macrocheilus immanis

Andrewes, 1920

http://species-id.net/wiki/Macrocheilus_immanis

Figs 3 , 11, 19 , 27, 28 , 45

Macrocheilus immanis Andrewes 1920: 498. Csiki 1932: 1574; Jedlička 1963: 469; Lorenz 2005: 512. Type locality: Myanmar (Taung-ngu), deposited in NHML.

#### Diagnosis.

Length 24.7 mm, width 8.3 mm. Labrum (Fig. 3) elongate, three pairs of equidistant setae on upper surface near margin; ligula thickened, apex slightly narrowed, slightly emarginate in middle of front margin, deeply depressed near apex to form a median channel, with five pairs of setae along sides, hollowed out above with a median carina; mentum (Fig. 11) with both tooth and lobes stout, tooth shorter than lobes, contracted after middle, irregularly setose on ventral surface on basal half; maxillary palpomere 4 (Fig. 19) dilated, densely setose; labial palpomere 4 dilated, palpomere 3 not dilated inwards. Elytal spots nearly square, large, near the middle, cover intervals 2–7.

Male genitalia. Median lobe of aedeagus strongly dilated and stout, strongly sinuate near paramere, straight towards apex, apical lamera elongate and narrowed at apex (Figs 27–28).

#### Remarks.

This species is similar to *Macrocheilus niger* and *Macrocheilus asteriscus* in having anterior setae of labrum obviously on upper surface, but differs from the other two species by the large and almost square elytral spots and stout median tooth of mentum.

#### Materials examined.

1 male, the holotype, labeled “Toungoo; Type; *Macrochilus immanis*, Type, Andr., H. E. Andrewes det.; *Macrochilus immanis* Andrewes, 1920”, deposited in NHML.

#### Distribution.

Myanmar.

### Macrocheilus niger

Andrewes, 1920

http://species-id.net/wiki/Macrocheilus_niger

Figs 4 , 12, 20 , 29, 30, 39 , 46

Macrocheilus niger Andrewes 1920: 499. Csiki 1932: 1574; Lorenz 2005: 512. Type locality: India (Nilgiri Hills), deposited in NHML.

#### Diagnosis.

Length 16.0–16.3 mm, width 6.0–6.2 mm. Labrum (Fig. 4) convex, with a short furrow on each side of base, narrowed and pointed in front, with three pairs of setae away from the margin; ligula thickened, apex truncate towards sides, apical margin slightly emarginate, deeply depressed towards base, with a small median impression near apex; mentum (Fig. 12) glabrous at base; median tooth shorter than lobes, rather narrow and with apex rather pointed, two pairs of setae at basal area; lateral lobes obtuse at apex. Palpi (Fig. 20) not dilated. Elytral spots transverse, close to the middle of interval 5, covering intervals 3–7.

Male genitalia. Median lobe stout, apical lamella short and round at apex (Figs 29–30).

Female genitalia. Gonocoxite subapically dilated, three setae on dorsal surface, sharp at apex (Fig. 39).

#### Remarks.

Similar to *Macrocheilus asteriscus*, but differs with the tooth of mentum plurisetose and elytral spot not cruciform.

#### Materials examined.

1 female, the holotype, labeled “type, 11, H. L. Andrewes, Nilgiri Hills, H. E. Andrewes Coll., B.M. 1945–97.; *Macrochilus niger* Type-, Andr., H. E. Andrewes det.; *Macrochilus niger* Andrewes, 1920”, deposited in NHML. 1 male and 1 female, “Dehra-Dun 1940-45, Kumaon Himalaya, Liesenfeldt leg.”, deposited in MNHN.

#### Distribution.

India (Nilgiri Hills, Malabar, Madras and Bombay) and Sri Lanka.

### Macrocheilus asteriscus

White, 1844

http://species-id.net/wiki/Macrocheilus_asteriscus

Fig. 47

Macrocheilus asteriscus White 1844: 422; Bates 1892: 389; Andrewes 1919: 180; 1920: 500; 1924: 470; 1930: 206; Csiki 1932: 1573; Wu 1937: 188; Jedlička 1963: 470; Hůrka 2003:407; Lorenz 2005: 512; Zhao and Tian 2010: 6. Type locality: China (Hongkong), deposited in NHML.
Planetes crucifer Redtenbacher 1867: 4. Type locality: China (Hongkong), deposited in NHML.

#### Remarks.

*Macrocheilus asteriscus* differs from other species in having cruciform elytral spots, the slender median tooth of mentum and the narrow apex of ligula.

#### Materials examined.

2 males, 1 female, “Hongkong” (MNHN); 2 males, “Hainan, Oct. 1979, Shaoming Zhuo leg.” (SCAU); 2 males, “Guangdong: Zhanjiang, Jul. 1982” (SCAU); 1 male, 1 female, “Guangdong: Zhanjiang, May, 1983” (SCAU). 1 male, 1 female, “Annam, Phuc-Son, Nov. to Dec., H, Fruhstorfer”, Central Vietnam (MNHN); 1 male, “Tonkin, P. Lemée, 1903–1906”, North Vietnam (MNHN); 1 male, “Laos. Mouhot” and “Janson Acq. 1884” (MNHN); 2 males, “Java, Preanger”, Indonesia (MNHN); 1 female, “Nilgherries” and “Ex. Musaeo H. W. Bates, 1892”, India (MNHN).

#### Distribution.

China, Vietnam, Laos, Myanmar, Indonesia and India.

### Macrocheilus vitalisi

Andrewes, 1920

http://species-id.net/wiki/Macrocheilus_vitalisi

Fig. 48

Macrocheilus vitalisi Andrewes 1920: 500; Andrewes 1930: 208; Csiki 1932: 1575; Wu 1937: 188; Jedlička 1963: 470; Hůrka 2003: 407; Lorenz 2005: 512; Zhao and Tian 2010: 7.

#### Remarks.

*Macrocheilus vitalisi* is similar to *Macrocheilus binotatus* from Sumatra, but as stated by Andrewes (1931), *Macrocheilus binotatus* differs from *Macrocheilus vitalisi* by “the dark colour, the elytral spot oblong and red; the upper surface generally is more coarsely and less densely punctuate, the genae are contracted more sharply to the neck, the sides of the prothorax are less sinuate behind, the elytral intervals more convex, with punctuation along the side more widely spaced and coarser”. In addition, according to our examination, the anterior seta of the labrum is closer to the apical margin in *Macrocheilus vitalisi* than in *Macrocheilus binotatus* and the 4th maxillary palpomere is cylindrically dilated in *Macrocheilus vitalisi*.

#### Materials examined.

1 female, the holotype, “China, Bowring 63·47*, 986 27/2/53” (NHML); 1 female, “Tonkin, Région de Hoa-Binh”, “Muséum Paris, 1932, A. de Cooman” (MNHN).

#### Distribution.

China, Laos, Vietnam, Borneo.

### Macrocheilus binotatus

Andrewes, 1931

http://species-id.net/wiki/Macrocheilus_binotatus

Figs 5 , 13, 21 , 40 , 49

Macrocheilus binotutats Andrewes 1931: 68. Csiki 1932: 1573; Lorenz 2005: 512. Type locality: Indonesia (Sumatra), deposited in LMN.

#### Diagnosis.

Length 14.0 mm, width 4.8 mm. Labrum (Fig. 5) with apex rounded and pointed, front setae on apical margin; mandibles covered by the labrum, sharp at apex; mentum (Fig. 13) glabrous at base, median tooth nearly as long as lobes, about five setae at basal half; maxillary palpomere 4 (Fig. 21) cylindrically dilated, labial palpomere 4 flat and dilated, labial palpomere 3 dilated inwards; ligula wide and rectangular, apical margin straight, with a wide median impression beneath apex, a seta on either side near apex. Elytral spots large and oblong, covering intervals 3–7 in the middle.

Female genitalia. Gonocoxite elongate, three setae on dorsal margin, sparsely setose on ventral surface; apex sharp (Fig. 40).

#### Remarks.

This species is similar to *Macrocheilus macromaculatus* having elytral spots oblong; and it differs from *Macrocheilus macromaculatus* in having the anterior setae of labrum on the apical margin.

#### Materials examined.

1 female, “Paggar Alam, Sumatra, J. Bouchaud”, deposited in MNHN.

#### Distribution.

Indonesia (Sumatra).

### Macrocheilus macromaculatus

Louwerens, 1949

http://species-id.net/wiki/Macrocheilus_macromaculatus

Macrocheilus macromaculatus Louwerens 1949: 51. Lorenz 2005: 512. Type Locality: Indonesia (Java), deposited in LMN.

#### Diagnosis.

Length 10.0 mm, width 3.0 mm. Labrum large, semicircular anteriorly, with a flat depression on each sides, setae running along the sides in two small furrows; palpi short and stout, truncate at apex; mentum with a long, narrow, sharp median tooth, lateral lobes a little longer. Elytral spots oblong and covers intervals 2–7.

The above description is after Louwerens (1949).

#### Remarks.

This species is similar to *Macrocheilus binotatus*, but the former is smaller in size and has the sides of elytra gently rounded behind, in contrast to the larger size and more sharply rounded hind region of elytra in *Macrocheilus macromaculatus*.

#### Distribution.

Indonesia (Java).

### Macrocheilus gigas

Zhao & Tian, 2010

http://species-id.net/wiki/Macrocheilus_gigas

Fig. 50

Macrocheilus gigas Zhao and Tian 2010: 8. Type Locality: China (Guangdong), deposited in SCAU.

#### Remarks.

*Macrocheilus gigas* is similar to *Macrocheilus parvimaculatus* and *Macrocheilus tripustulatus* in having anterior seta of the labrum on the apical margin and the ligula thickened at apex which distinguishes these three species from other species with two spots on each elytron. But *Macrocheilus gigas* differs from the other two species by wide apex of labrum and having median tooth of mentum sinuate near apex on lateral margin.

#### Materials examined.

1 male, the holotype, Guangdong: Zhanjiang, Jun.1983, deposited in SCAU; 1 male, Guangdong: Zhanjiang, Oct.1982, Zhichang Tan leg., deposited in SCAU; 2 males, Guangdong: Zhanjiang, July 1982, deposited in SCAU and MNHN.

#### Distribution.

China (Guangdong: Zhanjiang).

### Macrocheilus parvimaculatus

Zhao & Tian, 2010

http://species-id.net/wiki/Macrocheilus_parvimaculatus

Fig. 51

Macrocheilus parvimaculatus Zhao and Tian 2010: 9. Type Locality: China (Guangxi), deposited in SCAU.

#### Remarks.

This species is easily distinguished from other species of *Macrocheilus* by its strongly convex labrum, maxillary palpomere 4 dilated in apical half and with small spots on elytra.

#### Materials examined.

1 male, the holotype, Guangxi: Liuzhou: Luzhai, 26 May 1980, Shaozhou Ruan leg.; 1 male, same data as holotype. All specimens deposited in SCAU.

#### Distribution.

China (Guangxi).

### Macrocheilus tripustulatus

(Dejean, 1825)

http://species-id.net/wiki/Macrocheilus_tripustulatus

**Figure 52–61.**
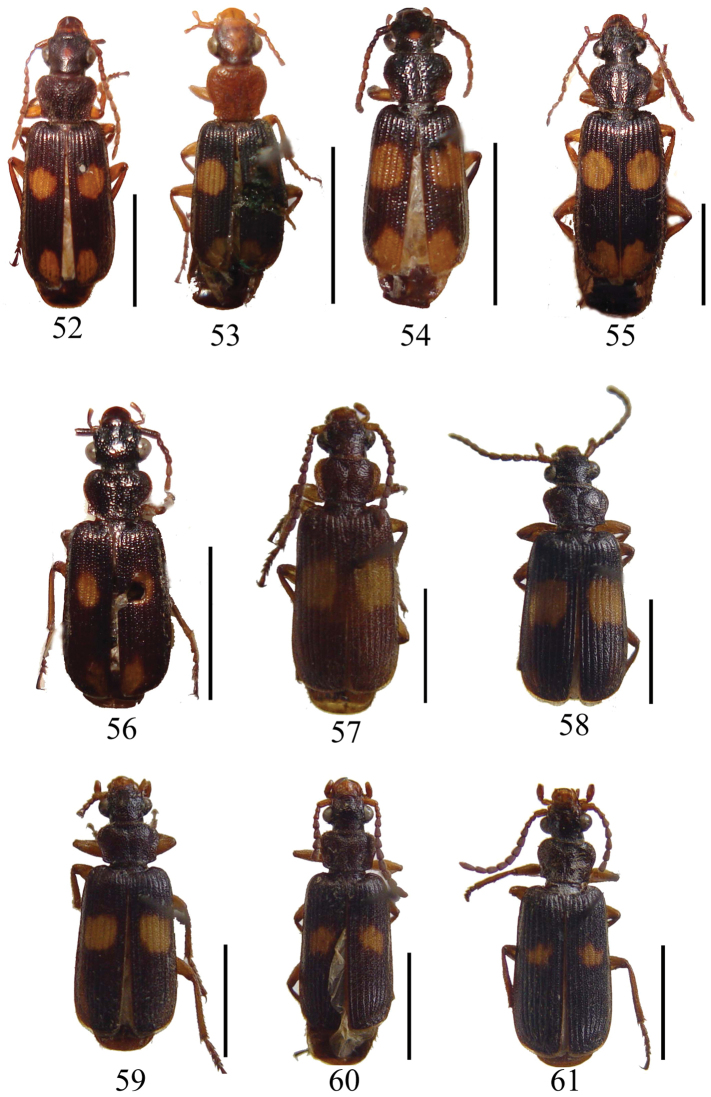
**52** *Macrocheilus tripustulatus* (holotype) **53** *Macrocheilus chaudoiri* (holotype) **54** *Macrocheilus nigrotibialis* (holotype) **55** *Macrocheilus bensoni* (male) **56** *Macrocheilus deuvie* (holotype) **57** *Macrocheilus fuscipennis* (holotype) **58** *Macrocheilus solidipalpis* (holotype) **59** *Macrocheilus cheni* (holotype) **60** *Macrocheilus quadratus* (holotype) **61** *Macrocheilus sinuatilabris* (holotype). Scale bar: 5.0 mm.

Figs 6 , 14, 22 , 31, 32, 41 , 52

Helluo tripustulatus Dejean 1825: 286 (syn. in Reiche 1842: 334). Reiche 1842: 334; Chaudoir 1872: 212; Heller 1900: 3; Andrewes 1919: 124; Andrewes 1920: 501; Csiki 1932: 1575; Lorenz 2005: 512. Type locality: Indonesia (Java), deposited in MNHN.

#### Diagnosis.

Length 12.0–12.5 mm, width 4.0–4.3 mm. Labrum (Fig. 6) strongly convex on anterior part, rounded and pointed in front, front seta on apical margin, intermediate one on upper surface close to margin; mandibles sharp at apex; mentum (Fig. 14) irregular and densely setose, both tooth and lobes stout, tooth shorter than lobes, densely setose at basal half; palpi slender, palpomere 4 (Fig. 22) not dilated, densely setose, rounded and truncate at apex, labial palpomere 3 not dilated, bisetose inside; ligula strongly thickened and dilated, deeply depressed at sides, apex rounded, slightly emarginate in middle with a seta on either side. Elytra with two pairs of spots, front spot rounded, covering intervals 3–7 in the middle, hind spot close to apical inner angles, nearly rectangular, covering intervals 1–5; apex roundly truncate.

Male genitalia. Median lobe dilated in the middle ventrally, apical lamella long, wide and round at apex (Figs 31–32).

Female genitalia: Gonocoxite slender, setose on ventral surface, three setae on dorsal side, apex sharp (Fig. 41).

#### Remarks.

*Macrocheilus tripustulatus* can be distinguished from *Macrocheilus parvimaculatus* by the slender maxillary palpomere 4, greater convexity of labrum and larger elytral spots.

#### Materials examined.

1 female, the holotype, “*tripustulatus* Dejean (non Fabr.), Java, Coll. Dejean, 3-*pustulatus* Wiedemann (*Helluo*). Fabr. (Brachinus), Western, Ex-Musaeo Chaudoir”; 1 male, “Java merid., 1500, 1891, H. Fruhstorfer.”; 1 male, “ Java”, “Ex-Musaeo Mniszech”; 1 female, “Carin Chebà, 900-1100 m, L. Fea, V Xii-88; Ex-Musaeo H.W.Bates 1892”. All specimens deposited in MNHN.

#### Distribution.

Myanmar and Indonesia (Java).

### Macrocheilus chaudoiri

Andrewes, 1919

http://species-id.net/wiki/Macrocheilus_chaudoiri

Fig. 53

Macrocheilus chaudoiri Andrewes 1919: 130; 1920: 502; 1924: 470; 1930: 207; Csiki 1932: 1573; Jedlička 1963: 470; Lorenz 2005: 512; Zhao and Tian 2010: 11. Type Locality: India, deposited in MNHN.
Acanthogenius trimaculatus sensu Chaudoir 1872: 171 (non Oliver). Type Locality: India (Decan), deposited in MNHN.
Macrocheilus ruficollis Heller 1923: 296; Andrewes 1926: 258. Type Locality: Philippines (Luzon), deposited in MDSG.

#### Remarks.

*Macrocheilus chaudoiri*, *Macrocheilus nigrotibialis*, *Macrocheilus bensoni* and *Macrocheilus deuvie* are similar in having the anterior seta of the labrum beneath the apex and two pairs of elytral spots. This species is distinct from the other three species in having the head and pronotum reddish brown and maxillary palpomere 4 strongly dilated.

#### Materials examined.

1 female, the holotype, “Ex-Musaeo Chaudoir; Macrocheilus Chaudoiri Andr., H.S. Andrewes det.; 3-maculatus Chaud., Deccan, Coll. Jeakes” (MNHN); 1 male, “Maissour, Sakrabail, IX 1897” (MNHN); 1 female, “Museum Paris, Cochinchine, Harmand 1872” (MNHN); 1 female, “Java” and “Museum Paris, Lakhon, Harmand 1878” (MNHN); 1 female, “Insl. Phiip.” and “Thorey” (MNHN).

#### Distribution.

China (Macao), Cambodia, Laos, Vietnam, the Philippines, Indonesia (Sumatra) and India.

### Macrocheilus nigrotibialis

Heller, 1900

http://species-id.net/wiki/Macrocheilus_nigrotibialis

Figs 7 , 15, 23 , 33, 34, 42 , 54

Macrochilus nigrotibialis Heller 1900: 2. Andrewes 1920: 497; Csiki 1932: 1574; Lorenz 2005: 512. Type locality: Indonesia (Sulawesi), deposited in MDSG.

#### Diagnosis.

Length 8.0–10.1 mm, width 3.0–3.5 mm. Labrum (Fig. 7) convex, arcuate at apex, front setae beneath apex, intermediate one on apical margin, hind one close to middle margin; mandibles sharp at apex; mentum (Fig. 15) setose at base; median tooth triangular and shorter than lobes, with two pairs of setae at base; lobes obtuse at apex; maxillary palpomere 4 (Fig. 23) roundly dilated, labial palpomere 4 triangular in shape and dilated, 3 not dilated and bisetose on inner sides; ligula thickened, apical margin arcuate inwards, deeply depressed at sides, with a median impression close to apex, outer apical angles rounded, with a seta on either of median impression close to apex. Elytra with front spot large, just before middle, almost rounded, covering intervals 3–7 and extended to small part of 2 and 8; hind spot on inner angles, more or less triangular, covering intervals 1–5.

Male genitalia. Median lobe slender, apical lamella round (Figs 33–34).

Female genitalia. Gonocoxite slender, arcuate, with three or four setae on dorsal side, apex slightly obtuse (Fig. 42).

#### Remarks.

This species differs from following two species in having the tibiae black, maxillary palpomere 4 more dilated, the median lobe of male genitalia stouter and the apical lamella more rounded.

#### Materials examined.

1 male, the holotype, “typus!”, “Drs. Sarasin N. Celebes Panot-Maimang”, “12623”, “Staatl. Museum fur Tierkunde. Dresden”, deposited in SNSD. 1 female, labeled “Nord Borneo Mont Kina Balu 5-8, 1903, John Waterstradt; *Macrochilus nigrotibialis* Heller, det. Andrewes”, deposited in MNHN.

#### Distribution.

Indonesia (Sumatra, Malaysia and Sulawesi).

### Macrocheilus bensoni

Hope, 1838

http://species-id.net/wiki/Macrocheilus_bensoni

Fig. 55

Macrocheilus bensoni Hope 1838: 166; Chaudoir 1872: 212; Bates 1892: 389; Heller 1900: 3; Andrewes 1919: 176, 202; Hůrka 2003: 407; Lorenz 2005: 512: Zhao and Tian 2010: 10. Type locality: India, deposited in NHML.
Carabus trimaculatus Olivier 1790: 347 (non Villers 1789); Andrewes 1919: 129, 176; 1920: 502; 1930, 208; Csiki 1932: 1574; Wu 1937: 188; Jedlička 1963: 470. Type locality: China, deposited in NHML.
Helluo quadrimaculata Guérin-Méneville 1840: 38; Chaudoir 1872: 212. Type locality: India, deposited in MNHN.
Helluo tripustulata sensu Guérin-Méneville 1843: 34 (non Dejean 1825); Andrewes 1923: 460. Type locality: China, deposited in MNHN.
Macrocheilus quadripustulatus Schmidt-Göbel 1846: 65. Type locality: Myanmar, deposited in NMP.
Acanthogenius infuscatus Bates 1892, 389; Andrewes 1920: 493. Type locality: Myanmar (Bhamo), deposited in MGI.

#### Remarks.

This species can be distinguished from *Macrocheilus chaudoiri* by the black head and pronotum and from *Macrocheilus nigrobibialis* by the slender maxillary palpomere 4.

#### Materials examined.

1 male, “North China, 1884, Janson” (MNHN); 1 male, “North India, Coll. Benson, Ex-Musaeo H.W. Bates, 1892” (MNHN); 1 male, “Ind. Angl., Coimbatore Dt, Siruveni, VI. 1937” (MNHN); 1 female, “Ind. Angl., Mysore” (MNHN); 1 male, “Guangdong: Zhanjiang, Oct.1982, Zhichang Tan leg.” (SCAU); 2 males, “Guangdong: Yingde, 27 Mar.2003, Danyang Zhao leg.” (SCAU and MNHN); 1 male, Guangxi, Dec.1983” (SCAU); 1 male, “Yunnan: Jinghong, Jul. 1985” (SCAU); 1 female, “Hainan: Diaoluoshan, 26 Nov.1963” (SCAU).

#### Distribution.

China (Fujian, Jiangxi, Guangdong, Guangxi, Guizhou, Yunnan, Hongkong, Hainan), Laos, Vietnam, Myanmar, India and Sri Lanka.

### Macrocheilus deuvie sp. n.

urn:lsid:zoobank.org:act:0181841F-FB7C-4A7D-9612-AB0BFEAD91F3

http://species-id.net/wiki/Macrocheilus_deuvie

Figs 8 , 16, 24 , 35, 36 , 56

#### Description.

Length 9.5 mm, width 3.5 mm.

Black; ligula, antennomeres 1-4, a spot on vertex, lateral margin of pronotum and legs reddish brown; sides of ligula, palpi, antennomeres 5-11 and elytral spots brown.

Head convex; neck short and punctate on dorsal surface; frontoclypeal sulcus faint, frontal foveae short and shallow; clypeus with apical margin truncate, two setae on each side of apical outer angels, a row of 6 setae along apical margin, irregularly setose basally on each side; labrum (Fig. 8) convex anteriorly, apical margin rounded, front setae beneath near apex, middle one just on apical margin, hind one close to middle margin; mandibles covered by labrum, sharp at apex; mentum (Fig. 16) irregularly setose and punctate at base, median tooth triangular and shorter than lobes, with a pair of setae at base, lobes obtuse at apex; maxillary palpi (Fig. 24) not dilated, labial palpomere 3 with two setae on inner side; ligula thickened, apex deeply and widely emarginated in middle.

Pronotum flat; widest before middle; faint median line, median and apical impression distinct, basal foveae deep; lateral margin round in front, strongly sinuate near base; hind angles nearly rectangular, with a small obtuse tooth and an emargination before tooth.

Elytra flat, striae with large, close punctures and setae; intervals convex, with two rows of regular punctures and setae, interval 8 wider than others and densely and irregularly punctate and setose; spots small, front spot rounded, just before middle, covering intervals 3–6, hind spot rhombic, on inner apical angles, covering intervals 1–5.

Male genitalia. Median lobe dilated in middle on ventral margin; apical lamella elongated, rounded at apex (Figs 35–36).

#### Remarks.

This species is very similar to *Macrocheilus bensoni*, but differs by it’s smaller size, curved labrum at anterior part, clypeus glabrous on middle, pronotum with lateral margin strongly sinuate near base, and male genitalia dilated on ventral margin.

#### Type material.

1 male, the holotype, “Philippines, Bohol. Ch. Semper”, deposited in MNHN.

#### Etymology.

This species is named in honor of Dr. Thierry Deuve (MNHN), a well known carabidologist.

#### Distribution.

The Philippines.

### Macrocheilus fuscipennis

Zhao & Tian, 2010

http://species-id.net/wiki/Macrocheilus_fuscipennis

Fig. 57

Macrocheilus fuscipennis Zhao and Tian 2010: 12. Type locality: China (Guangxi), deposited in SCAU.

#### Remarks.

This species is similar to *Macrocheilus solidipalpis* and *Macrocheilus cheni* in having the larger elytral spots, but easily distinguishable from them by the labrum without additional setae, mandibles obtuse at apex, median tooth of mentum with lateral margin not sinuate and body brownish.

#### Materials examined.

1 male, the holotype, “Guangxi: Napo, 10 Oct.1970, by light trap”.

#### Distribution.

China (Guangxi: Napo).

### Macrocheilus solidipalpis

Zhao & Tian, 2010

http://species-id.net/wiki/Macrocheilus_solidipalpis

Fig. 58

Macrocheilus solidipalpis Zhao and Tian 2010: 13. Type locality: China (Guangxi), deposited in SCAU.

#### Remarks.

This species is similar to *Macrocheilus cheni* but easily distinguishable from the latter by the presence of additional setae between the anterior and anterior setae, the median tooth of the mentum with sinuate lateral margins in middle (sinuate on apical one-third in *Macrocheilus cheni*), the median lobe larger, and the apical lamella long and narrowed towards apex.

#### Materials examined.

1 male, the holotype, “Guangxi: Dibei, Oct.1980, Xiuzhen Mao leg., by light trap”.

#### Distribution.

China (Guangxi: Dibei).

### Macrocheilus cheni

Zhao & Tian, 2010

http://species-id.net/wiki/Macrocheilus_cheni

Fig. 59

Macrocheilus cheni Zhao and Tian 2010: 14. Type locality: China (Guangxi), deposited in SCAU.

#### Remarks.

This species is similar to *Macrocheilus solidipalpis* and the differences from the latter were mentioned above.

#### Materials examined.

1 male, the holotype,“Guangxi: Tengxian, Oct.1980”.

#### Distribution.

China (Guangxi: Tengxian).

### Macrocheilus quadratus

Zhao & Tian, 2010

http://species-id.net/wiki/Macrocheilus_quadratus

Fig. 60

Macrocheilus quadratus Zhao and Tian 2010: 16. Type locality: China (Guangxi), deposited in SCAU.

#### Remarks.

This species is easily distinguished from other species by the shape of pronotum.

#### Materials examined.

1 male, the holotype, labeled “Guangxi: Cangwu, 1980, in paddy field”.

#### Distribution.

China (Guangxi: Cangwu).

### Macrocheilus sinuatilabris

Zhao & Tian, 2010

http://species-id.net/wiki/Macrocheilus_sinuatilabris

Fig. 61

Macrocheilus sinuatilabris Zhao and Tian 2010: 17. Type locality: China (Guangxi), deposited in SCAU.

#### Remarks.

*Macrocheilus sinuatilabris* is similar to *Macrocheilus quadratus* in having three pairs of setae on the labrum and small elytral spots. However, it differs from *Macrocheilus quadratus* in having smaller elytral spots, the median tooth of the mentum obtuse at its apex, the rounded apex of the labrum, the close positioning of front and intermediate labral setae, and the slender median lobe of the aedeagus.

#### Materials examined.

1 male, the holotype, labeled “Guangxi: Fenghuangcheng, Sep.1981, by light trap”.

#### Distribution.

China (Guangxi: Fenghuangcheng).

##### Distribution of *Macrocheilus* in the Oriental Region

**Figure 62.**
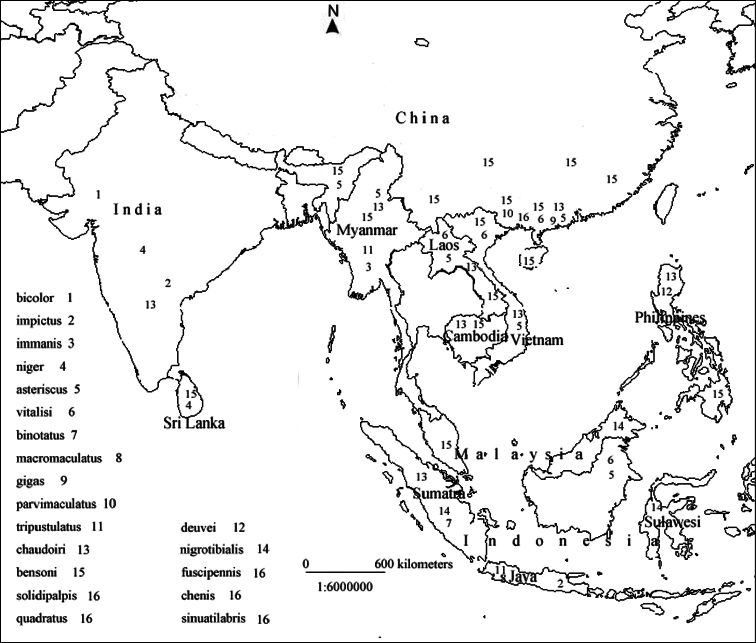
Distribution map of *Macrocheilus* in the Oriental Region.

The zoogeographical pattern of *Macrocheilus* is illustrated in Figure 62. Most species are distributed in limited small areas, but three are widespread, occurring in many countries: *Macrocheilus asteriscus* in China, Vietnam, Laos, Myanmar, Indonesia and India; *Macrocheilus chaudoiri* in China, Cambodia, Laos, Vietnam, the Philippines, Sumatra and India; and *Macrocheilus bensoni* in China, Laos, Vietnam, Myanmar, India and Sri Lanka. At present the *Macrocheilus* faunas of Thailand and Nepal remain unknown.

## Supplementary Material

XML Treatment for Macrocheilus

XML Treatment for Macrocheilus bicolor

XML Treatment for Macrocheilus impictus

XML Treatment for Macrocheilus immanis

XML Treatment for Macrocheilus niger

XML Treatment for Macrocheilus asteriscus

XML Treatment for Macrocheilus vitalisi

XML Treatment for Macrocheilus binotatus

XML Treatment for Macrocheilus macromaculatus

XML Treatment for Macrocheilus gigas

XML Treatment for Macrocheilus parvimaculatus

XML Treatment for Macrocheilus tripustulatus

XML Treatment for Macrocheilus chaudoiri

XML Treatment for Macrocheilus nigrotibialis

XML Treatment for Macrocheilus bensoni

XML Treatment for Macrocheilus deuvie

XML Treatment for Macrocheilus fuscipennis

XML Treatment for Macrocheilus solidipalpis

XML Treatment for Macrocheilus cheni

XML Treatment for Macrocheilus quadratus

XML Treatment for Macrocheilus sinuatilabris
